# The Tragedy of Alzheimer’s Disease: Towards Better Management via Resveratrol-Loaded Oral Bilosomes

**DOI:** 10.3390/pharmaceutics13101635

**Published:** 2021-10-07

**Authors:** Haidy Abbas, Heba A. Gad, Mohamed A. Khattab, Mai Mansour

**Affiliations:** 1 Department of Pharmaceutics, Faculty of Pharmacy, Damanhour University, Damanhour 22511, Egypt; Haidyabbas@pharm.dmu.edu.eg; 2 Department of Pharmaceutics and Industrial Pharmacy, Faculty of Pharmacy, Ain Shams University, Cairo 11566, Egypt; 3 Department of Cytology and Histology, Faculty of Veterinary Medicine, Cairo University, Cairo 12211, Egypt; mabdelrazik@cu.edu.eg

**Keywords:** Alzheimer, resveratrol, bilosomes, oxidative stress, blood-brain barrier

## Abstract

Alzheimer’s disease (AD) is a neurodegenerative disease where oxidative stress plays a major role as a key pathologic factor. The study aims to develop resveratrol (RES)-loaded bilosomes for oral use, aiming to enhance RES bioavailability. RES-loaded bilosomes were prepared using the thin-film hydration technique. The effect of different formulation variables *viz*. the number of extrusion cycles, drug concentration and the effect of pH of the medium and cholesterol addition on the physicochemical properties of the prepared bilosomes was investigated. Results revealed the successful entrapment of RES into bilosomes. An optimized formula was selected, showing the lowest particle size (189 ± 2.14), acceptable PDI (0.116) and entrapment efficiency (76.2 ± 1.36). In vivo studies on a streptozotocin-induced animal model of AD showed the preeminence of bilosomes over traditional drug suspension to enhance mice memory via Y-maze and Morris water maze tests. Moreover, mice treated with the optimized formula exhibited decreased COX2, IL-6, amyloid-beta peptide and Tau protein levels compared to the drug suspension. Immuno-histochemical analysis revealed a significant decrease of glial fibrillary acidic protein values and microglial cell count in mice treated with bilosomes. Finally, it could be advocated that RES-loaded bilosomes could be a promising drug delivery system to control AD.

## 1. Introduction

Alzheimer’s disease (AD) is a major predicament that affects a high percentage of the elderly population. It represents the principal cause of dementia and is one of the leading causes of death, increasing incidence among other neurodegenerative diseases. It is characterized by continuous deterioration of social and intellectual functions, memory loss and the cognitive deficiency [1]. The main mechanism involved in AD is still unknown. However, five main hypotheses were employed, including the β-amyloid protein cascade hypothesis, the Tau protein hypothesis, the cholinergic hypothesis, mitochondrial cascade and excitotoxicity hypothesis [2].

Prior researches substantiate the belief that ageing and neurodegenerative diseases are closely related. Ageing is accompanied by impairment in the endogenous antioxidant system with a subsequent rise in the accumulation of the reactive oxygen species (ROS). This ROS emanates oxidative stress with many harmful effects embracing DNA damage, mitochondrial dysfunction, apoptosis and inflammatory reaction [3]. So far, AD is intimately linked to oxidative stress, which causes a significant disruption in the balance between anti- and pro-inflammatory reactions inducing neuron inflammation. Consequently, AD is accompanied by elevated levels of oxidative stress biomarkers such as catalase (CAT), superoxide dismutase (SOD), glutathione (GSH) peroxidase and GSH reductase in the brain and inflammatory biomarkers such as cyclooxygenase (COX) and interleukins such as IL-6, IL-1β and TNF-α [4]. COX2 and IL-6 represent two oxidative biomarkers that had been related to neurodegenerative diseases due to their involvement in the inflammatory response with subsequent increase in neurons damage [4].

Moreover, the pathological findings of AD include extracellular β-amyloid precipitation, hyperphosphorylation of Tau (p-Tau) protein and neurofibrillary tangle accumulation in the brain tissues that induce neuro-inflammation and result in neurotoxicity [5]. Other manifestations include loss of synaptic plasticity, cholinergic dysfunction with decreased choline uptake and acetylcholine release.

Prior investigations are directed towards controlling the symptoms of AD where they have queried diverse approaches to identify possible prophylactic and therapeutic interventions. Management of AD may depend on acetylcholinesterase inhibitors such as tacrine, galantamine, donepezil, physostigmine and rivastigmine to increase the acetylcholine levels in the cholinergic synapses. Other modalities include the use of drugs that decrease β-amyloid and Tau proteins accumulation in the brain with anti-inflammatory and antioxidant effects such as polyphenols [6,7].

The use of natural products is widely applied for the management of different chronic and progressive diseases. Polyphenols are extensively used for the prevention of age-related diseases owing to their anti-inflammatory and neuroprotective effects. Among the known polyphenols, resveratrol (RES) is widely present in fruits, especially grapes, berries and nuts. RES has many therapeutic activities, including anti-inflammatory, antioxidant, anti-proliferative, in addition to its neuro-, cardio- and hepatoprotective effects [8].

RES has proved an eloquent effect in AD treatment due to its antioxidant, anti-inflammatory and anti-apoptosis effects [9,10] and its ability to bind to amyloid beta-peptide 1–42 (Aβ1-42). In addition to its ability to split Aβ1-42 into small fragments, preventing its aggregation in the brain and inhibiting the tau pathway [11].

Oral administration endures being the most preferred route for drug administration along with enhanced patient acquiescence. However, RES has many drawbacks associated with its oral use, including its low solubility, poor bioavailability, chemical instability, hepatic metabolism and rapid elimination. Encapsulation of RES into nanocarriers may overcome the obstacles of instability and solubility and hence increasing its bioavailability [12].

Attempts to deliver RES for the treatment of AD using nanocarriers include solid lipid nanoparticles [13], selenium nanoparticles [14], lipid core nanocapsules [15], transferosomes and nanoemulsions [16], polysorbate 80-coated poly (lactide) nanoparticles [17] and polymeric micelles [18].

Bilosomes are modified liposomes via the inclusion of bile salts to overcome some of the liposome drawbacks. Liposomes can partially protect the encapsulated molecules from the harsh environment of the GIT. However, the bile salts of the GIT bring vesicular deformation and lysis with subsequent early release of the entrapped drugs. The incorporation of bile salts in the vesicles’ membrane stabilizes the vesicles due to the repulsive effect of GIT bile salts and their resistance to digestive enzymes. Bilosomes are characterized by their higher GIT stability and permeability, which allows the delivery of large hydrophilic molecules like proteins, peptides and vaccines [19]. Moreover, bile salts included within the bilosomes such as sodium glycocholate, sodium deoxycholate (SDC) and sodium taurocholate act as penetration enhancers to increase the oral bioavailability of drugs with low aqueous solubility and permeability [20].

The present work aims to investigate the ability of bilosomes to inflate resveratrol GIT permeability and bioavailability. Full in vitro characterizations of the prepared RES-loaded bilosomes was performed. The aim is to obtain a formula with favorable particle size, high drug entrapment efficiency and fast drug release. In addition, the study aims to evaluate the therapeutic virtue of the optimized RES-loaded bilosomes in the treatment of Alzheimer’s disease in a mice model by studying the levels of oxidative stress biomarkers, amyloid-beta peptide and Tau protein levels, besides the histological examination, immuno-histochemical analysis and behavioral assessment.

## 2. Materials and Methods

Resveratrol (RES) (trans, 98% content) was purchased from Behr GmbH., Stuttgart, Germany. LIPOID S100 (SPC soybean phosphatidylcholine) was a kind gift from LIPOID Co., Ludwigshafen, Germany. Cholesterol (CH), and sodium deoxycholate (SDC) was obtained from Sigma Aldrich, Darmstadt, Germany. Sodium chloride, potassium chloride, potassium dihydrogen phosphate, ethanol and ether were purchased from Prolabo, Adwic, El-Nasr Pharmaceutical Co., Cairo, Egypt. All other chemicals were of analytical grade.

### 2.1. Preparation and Optimization of Different Vesicles

Bilosomes were prepared using the thin-film hydration technique [21]. In brief, SPC dissolved in ethanol was introduced in a rotary evaporator at 40 °C to evaporate ethanol and form a thin film. The thin film was hydrated using 0.1 M phosphate buffer saline (PBS) containing SDC and RES with continuous shaking until the formation of the vesicles. Preparations were left to mature overnight at 4 °C. Optimization of RES-loaded bilosomes was done by varying factors such as the number of extrusion cycles, drug concentration, the pH of the medium and the effect of cholesterol addition. Extrusion was performed by pressing the syringe against 0.2 um polycarbonate filter at 30 °C or 60 °C for CH containing preparations. Table 1 shows the composition of different RES-loaded bilosomes.

### 2.2. Physicochemical Characterization

#### 2.2.1. Particle Size, Polydispersity Index and ZP

Measurement of particle size and polydispersity index of RES-loaded bilosomes was determined after suitable dilution with PBS. Measurements were performed using Malvern zeta sizer Nano ZS (Malvern Instruments, Malvern, UK) with a dynamic light scattering particle size analyzer at 25 °C [22]. Zeta potential was determined using the same device with using folded capillary zeta cells. Measurements were done in triplicates.

#### 2.2.2. Entrapment Efficiency

The percent entrapment efficiency (%EE) was determined using the direct method [23]. Formulations were subjected to centrifugation at 15,000 rpm for 1 h to separate the free unentrapped drug, and the clear supernatant was discarded. The separated nanoparticles were sonicated in ethanol for 10 min. Solutions were filtered with a syringe filter (pore size: 0.4 μm) (Millex-LG, Millipore Co., Billerica, MA, USA). The amount of RES entrapped was quantitatively assessed spectrophotometrically using a UV spectrophotometer (Shimadzu 2401/PC, Tokyo, Japan) at 303 nm.

The encapsulation efficiency percentage was calculated from the following equation:

EE % = (Amount of encapsulated drug)/(Total amount of drug) × 100

#### 2.2.3. In Vitro Drug Release Study

Drug release from loaded bilosomes (formula showing favorable particle size, PDI and highest entrapment efficiency) and RES suspension was assessed using the dialysis bag method (Visking^®^ 36/32, 28 mm, MWCO 12,000–14,000; Serva, Heidelberg, Germany). The dialysis bag (5 cm) containing 0.2 mL of the preparation was submerged in a release medium made up of 15 mL of 0.1 M PBS (pH 7.4), maintained at 37 ± 0.5 °C and 100 rpm thermostated shaking water bath. Samples were taken at the following intervals (15, 30, 45, 60 and 120 min) and measured spectrophotometrically at 303 nm against blank (0.1 M PBS). The experiment was repeated in triplicate [21].

#### 2.2.4. Transmission Electron Microscopy (TEM)

The selected RES-loaded bilosomes were examined using TEM (JEM-1010, JEOL Ltd., Tokyo, Japan) for morphological assessment. Samples were first vortexed and diluted. Few drops were placed on a copper grid and left to dry for few minutes at room temperature before TEM examination [22].

### 2.3. In Vivo Studies

#### 2.3.1. Animals

Adult male Albino mice weighing from 20 to 25 g obtained from the animal house of the National Research Center, Cairo, Egypt, were included in this study. The mice were kept in plastic cages in the air-controlled room at 25 °C allowing a 12 h light and dark cycle and free access to food and water. The study was conducted after being approved by “Experiments and Advanced Pharmaceutical Research Unit” of the Faculty of Pharmacy, Ain Shams University, Cairo, Egypt, and National Institutes of Health guidelines for the use and care of Laboratory animals (approval number 23).

#### 2.3.2. Groups

Animals were randomly divided into four groups (each group contained eight mice). Group 1 was normal mice with no AD induction and no treatment (served as normal), Group 2 served as a negative control, while Group 3 served as the positive control and Group 4 served as the test group for the selected bilosomes. For the induction of AD, Groups 2, 3 and 4 received intra-cerebroventricular (ICV) injection of Streptozocin (STZ) (3 mg/kg) once on the first day, followed by oral administration of 0.9% saline, RES suspension (10 mg/kg/day) and RES-loaded bilosomes (10 mg/kg/day) for Groups 2, 3 and 4 respectively for 21 days once daily. All behavioral, biochemical, histological and immuno-histochemical assessments were conducted on day 21.

#### 2.3.3. Induction of AD

For the induction of AD, mice were anaesthetized with ether for ICV administration that was performed under aseptic conditions. After confirming anesthesia, mice heads were kept fixed by applying downward pressure above the ears. STZ was injected by direct insertion of the needle into the lateral cerebral ventricle, where 3 µL were delivered slowly at a rate of 1 µL/min [24].

#### 2.3.4. Behavioral Assessment

##### Y-Maze Test

The Y-maze test is a behavioral test that evaluates short-term memory by measuring the ability of mice to explore the new environment. The test is performed using a Y-shaped maze with three metallic arms (35 cm long, 25 cm high and 10 cm wide) placed at 120°. Normal rodents usually prefer to enter a new arm of the maze rather than the recently visited one. The test was carried out on two successive days. The first day was assigned for mice training, where each mouse was introduced into the center of the maze and allowed to explore the maze for 10 min freely. The mice should show a greater tendency to enter the non-visited arm. On the next day, the number of entries into each arm and the number of alterations with their sequences were recorded for 10 min to calculate the percentages of spontaneous alterations. The percentage of spontaneous alternation behavior was calculated as the ratio of actual alternations to possible alternations multiplied by 100 [25].

##### Morris Water Maze (MWM) Test

The MWM test is widely used in behavioral neuroscience to study the spatial learning and memory of animals. A large circular pool made of stainless steel (150 cm in diameter and 60 cm in height) half-filled with water and maintained at room temperature was employed in the study. The pool was divided arbitrarily into four equal quadrants. A fixed submerged platform (10 cm width, 28 cm in height) was placed inside the target quadrant of this pool 2 cm below the water surface. Normal animals have the ability of quick learning to swim towards the platform. At the same time, the acquisition phase for each mouse was carried out for four days with two consecutive trials where a gap of 15 min between each trial was employed. The maximum time allowed for each trial was 120 s. On the fifth day, the mice were subjected to a probe-trial phase after platform removal. The time spent by each mouse to locate the place of the removed platform was recorded and taken as an index of retrieval or memory. The mean escape latency (MEL) time is the time spent by each mouse to find the hidden platform during each trial performed over the four testing days.

**Figure 1:**
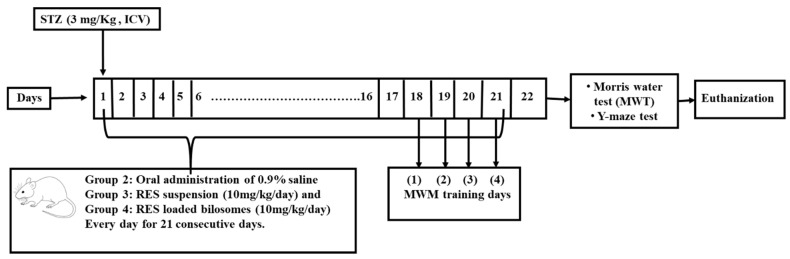
A schematic diagram for the experimental design showing Alzheimer’s disease induction by intra-cerebroventricular injection (ICV) of Streptozocin (STZ) (3 mg/kg) on the first day, followed by oral administration of 0.9% saline, Resveratrol (RES) suspension (10 mg/kg/day) and RES-loaded bilosomes (10 mg/kg/day) for Groups 2, 3 and 4 respectively for 21 days. Each group contained eight mice. All behavioral, biochemical, histological and immuno-histochemical assessments were conducted on day 21.

After performing the behavioral tests, animals were sacrificed by cervical dislocation after being anaesthetized using thiopental sodium (50 mg/kg i.p). The whole brain was removed and washed with ice-cold saline. The isolated brains from each group were divided into two sets for neurochemical assay, histological and immuno-histochemical examination. Figure 1 shows a schematic diagram for the experimental design.

#### 2.3.5. Neurochemical Assay

Brain samples for neurochemical assay were homogenized with normal saline using a homogenizer mixer (IKA, T 25 digital ULTRA-TURRAX, Staufen, Germany). The homogenate was centrifuged for 10 min at 4 °C, and the supernatant was collected and stored at −80 °C for further analysis.

##### IL-6 Assessment

IL-6, one of the potent pro-inflammatory cytokines, was quantitatively determined in the brain homogenates from all groups using the mouse IL-6 (Interleukin 6) ELISA Kit (MBS 2,508,516 96T, Mybiosource kit, San Diego, CA 92195-3308, USA) according to the instructor’s protocol.

##### COX2 Assessment

COX2 was quantitatively determined in the brain homogenates from all groups using the mice Simple Step ELISA^®^ Kit (ab210574, Abcam kit, Cambridge, CB2 0AX, UK) according to the instructor’s protocol.

##### Amyloid-Beta Peptide 1–42(Aβ1-42) Assessment

The levels of Aβ1-42 in the brain homogenates were determined using mouse Amyloid beta-peptide 1–42(Aβ1-42) ELISA^®^ Kit (MBS265825m Mybiosource kit, San Diego, CA 92195-3308, USA) that employs the Double Antibody Sandwich technique.

##### Tau Protein Assessment

The levels of tau, the microtubule-associated protein, was determined by colorimetry in the brain homogenates using Mouse Tau ELISA^®^ Kit (NBP2-81260, Novus kits, Centennial, CO 80112, USA) according to the instructor’s protocol.

#### 2.3.6. Histological Examination

Mice brain tissue samples were dissected, flushed and fixed in 10% neutral buffered formalin for 72 h. Samples were processed in serial grades of ethanol, cleared in xylene, infiltrated and embedded into Paraplast plus tissue embedding media (Leica biosystems, Buffalo Grove, IL 60089 United States). Serial sagittal brain sections of 4 μm thick were cut by rotatory microtome to demonstrate the hippocampal regions in different samples and then mounted on glass slides. Tissue sections were stained by Hematoxylin and Eosin as a general staining method for microscopic examination and stained by Toluidine blue stain to demonstrate damaged and intact neurons, then examined by using a light microscope. All standard procedures and protocols for samples fixation and staining were performed according to Culling, 2013 [26].

#### 2.3.7. Immuno-Histochemical Staining and Analysis

Immuno-histochemical staining for glial fibrillary acidic protein (GFAP) and IbA-1 and β-amyloid was conducted according to the manufacturer’s protocols and directions. Antigen Retrieved Brain tissue sections endogenous peroxidase was blocked by 3% hydrogen peroxide in methanol for 15 min followed by overnight incubation at 4 °C with the primary antibodies Anti-Glial Fibrillary Acidic Protein, by using a monoclonal antibody kit (Cat. No. 13-0300-Thermo-scientific Co. Waltham, MA, USA) 1:100), Anti-Iba1 antibody (ab108539-Abcam-1:100) and Anti-beta Amyloid antibody (ab201060-Abcam-1:500)was done, followed by washing with 3× PBS then incubation of the secondary antibody Horseradish Peroxidase (HRP) Envision kit (DAKO, Carpenteria, CA, USA) for 20 min, followed by 3× PBS washing and incubation with diaminobenzidine for 10 min. Then, counterstaining with Mayer’s hematoxylin was performed, followed by dehydration and clearing in xylene. Tissue sections were then covers-lipped for microscopic examination.

##### Immuno-Histochemical Quantitative Analysis

Six random non-overlapping fields were scanned and analyzed for determining the positive mean area percentage of immuno-histochemical expression levels of GFAP and Beta amyloid as well as mean numbers of IbA-1/++ microglial cells in each immuno-stained tissue section CA3 subregions. All morphological examinations and quantitative analysis were recorded using Leica Application system modules for histological analysis (Leica Microsystems GmbH, Wetzlar, Germany).

### 2.4. Statistical Analysis

All in vitro data are expressed as a mean of three replicates ± standard deviation of the mean. Statistical significance of all in vitro and in vivo results were analyzed using GraphPad Prism 8.3.8. One-way analysis of variance (ANOVA) followed by the Tukey–Kramer post hoc test were used to compare the groups’ differences. The differences between the groups were considered statistically significant if *p* was < 0.05.

## 3. Results and Discussion

### 3.1. Physicochemical Characterization of RES-Loaded Bilosomes

#### 3.1.1. Effect of Extrusion Cycles

Particle size is (PS) an important parameter that controls the penetration of the vesicles within the intestinal tissue. The smaller the vesicular size, the higher the penetration rate is. The effect of different variables on the PS, polydispersity index (PDI) and zeta potential (ZP) of RES-loaded bilosomes is shown in Table 1. All the prepared RES-loaded bilosomes showed PS ranging from 189 ± 2.14 to 381 ± 2.6 nm. This nano-range PS is due to the effect of SDC that acts as a surfactant decreasing the membrane surface tension followed by a decrease in PS.

Extrusion cycles ranging from one to four cycles were employed for the preparation of RES-loaded bilosomes. As revealed from the results, varying the number of extrusion cycles while keeping other variables constant significantly affected the PS and PDI. Increasing the number of extrusion cycles from 1 to 3 is accompanied by a significant decrease (*p* < 0.05) in PS and PDI values; however, further increase in the number of extrusion cycles to four resulted in a non-significant decrease (*p* > 0.05) in PS and a significant increase in PDI. Our results are following Elnaggar et al. [20], who proved an optimum number of extrusion cycles after which no significant effect on PS was observed.

Zeta potential results show that all RES-loaded bilosomes carry a negative charge that may be attributed to the presence of a carboxylic group in the bile salt. SDC contributes to the formation of the lipid bilayer rather than being adsorbed on the surface of the vesicle [27,28]. Increasing the number of extrusion cycles was accompanied by a non-significant (*p* ˃ 0.05) decrease in the ZP.

The %EE of RES-loaded bilosomes are presented in Table 1. As revealed, the different studied factors have a significant (*p* < 0.05) effect on the %EE that range from 16.3% ± 1.56 to 76.2% ± 1.36. The investigated extrusion cycles ranged from one to four cycles, where the usage of three cycles lead to nanoparticles with the highest EE% of RES. Based on the previous results, B3 that showed the highest %EE of the drug along with a favorable size (198 ± 1.45 nm), was selected to study the effect of drug loading.

#### 3.1.2. Effect of Drug Loading

Different concentrations of RES, ranging from 5 to 30 mg/mL, were tested on RES-loaded bilosomes. Being a hydrophobic molecule, RES was expected to be located within the lipid bilayer rather than within the aqueous core of the vesicles. It is observed from Table 1 that increasing the drug concentration from 5 to 10 mg is associated with a significant increase (*p* < 0.05) in PS and PDI values. This increase may be assigned to the incorporation of RES in the membrane bilayer with a subsequent increase in the diameter of the vesicles. In addition, increasing the drug concentration to 20 and 30 mg resulted in a slight non-significant (*p* ˃ 0.05) increase in PS, associated with high PDI values ˃ 0.4, indicating more than one population in the dispersion. The obtained results may be assigned to the inability of the prepared vesicles to accommodate more drug within the lipid bilayers upon increasing the drug concentration from 10 mg to 20 mg.

As previously demonstrated, the negative charge of the prepared bilosomes may be attributed to the presence of bile salts. Accordingly, increasing the drug concentration from 5 to 30 mg was accompanied by a non-significant (*p* ˃ 0.05) change in the zeta potential values of RES-loaded bilosomes.

Increasing the drug concentration from 5 to 10 mg is associated with a significant increase in %EE (*p* < 0.05), which may be attributed to the saturation of the media with REV that drives more of the drug to be entrapped into bilosomes [29]. However, a further increase in drug concentration to 20 and 30 mg/mL caused significant decrease to 54 ± 2.52 and 42 ± 1.96%, respectively. Based on the previous results, drug loading of 10 mg (B5) was selected to study the effect of pH of the medium and CH addition.

#### 3.1.3. Effect of the pH of the Medium and Cholesterol Addition

Results in Table 1 show that decreasing the pH of the medium is accompanied by a significant decrease (*p* < 0.05) in PS and PDI values and a non-significant increase (*p* > 0.05) in ZP values. However, the addition of CH is accompanied by a significant decrease in PS and a non-significant change in PDI and ZP values. This change may be attributed to the effect of CH on stabilizing the lipid bilayer. The presence of CH within the vesicles causes a reduction in the surface tension with the subsequent formation of smaller particles. In addition, CH increases the vesicles’ rigidity and particle stability with a subsequent decrease in particle fusion.

Decreasing the pH of the medium is accompanied by a significant change in the %EE, where pH 3 showed a higher %EE of 72.3 ± 1.87% than pH 7.4 of 67 ± 1.56%. This change could be assigned to the effect of changing the pH on the ionization of RES and SDC. RES has three acidic dissociation constants (pKa1, 2, 3 = 8.8, 9.8 and 11.4 respectively); therefore, at low pH values, protonation of the three hydroxyl groups occurs, and the drug exists in a unionized form that leads to decreasing the repulsive forces with SDC and higher %EE [30].

The presence of CH is associated with a significant (*p* < 0.05) increase in %EE from 72.3 ± 1.87% to 76.2 ± 1.36%, which is accredited to the ability of CH to enhance the hydrophobicity, rigidity and stability of the lipid bilayer with a subsequent decrease in the drug leakage from the vesicles [23,29,31].

### 3.2. In Vitro Drug Release

**Figure 2:**
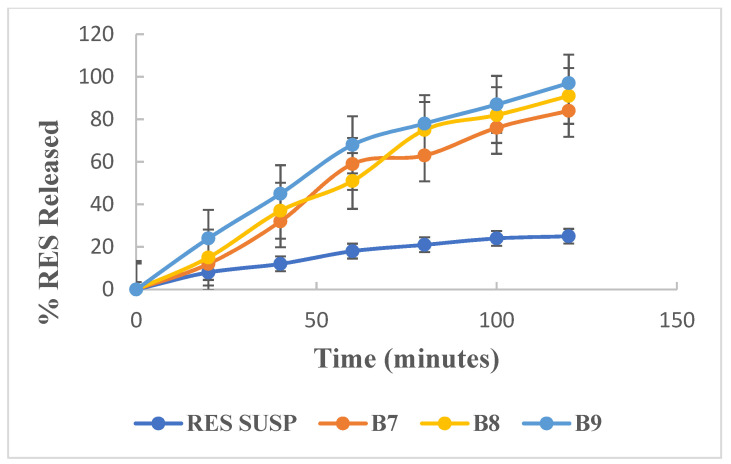
In vitro release study of resveratrol from different formulations in phosphate buffer saline pH (7.4) using the dialysis bag method.

The effect of changing the pH of the medium and the addition of CH on the RES release rate from the prepared bilosomes was investigated. Figure 2 shows the results of the invitro release studies of the selected formulae (B7, B8 and B9) in comparison to RES suspension. RES release from suspension is characterized by a slow rate with only 18% and 25% released after 1 and 2 h, respectively, indicating incomplete drug release. In contrast, all RES-loaded bilosomes show gradual and almost complete release profiles with RES percent release ranging from 81% to 91% after 2 h, while RES-loaded bilosomes formula containing cholesterol shows a 97% RES release after 2 h, indicating the ability of the prepared vesicles to enhance RES release.

Based on the previous results, B9, showing the highest %EE and fastest drug release and favorable particle size, was selected for further study.

### 3.3. TEM

**Figure 3:**
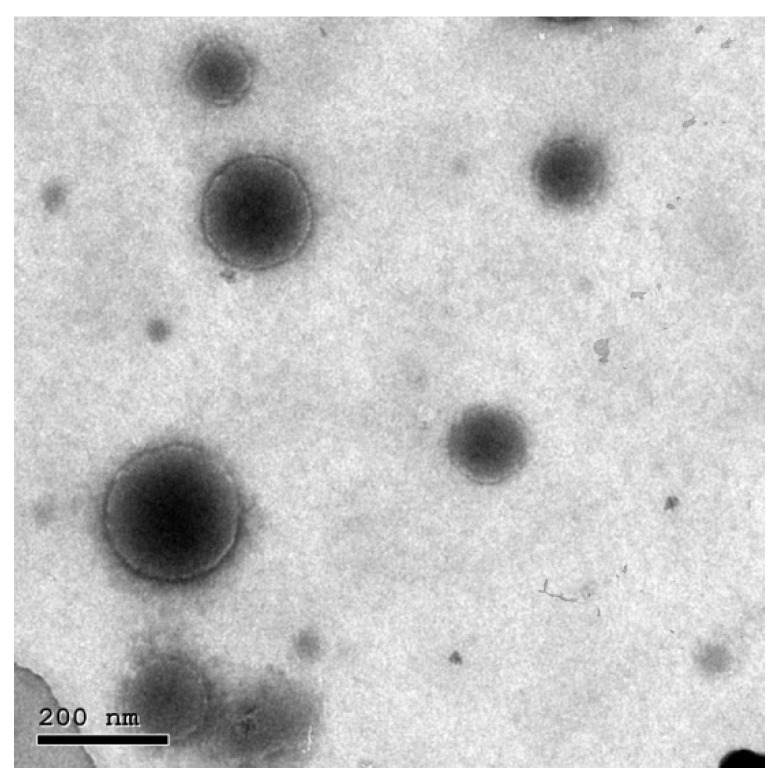
TEM micrograph of representative resveratrol-loaded bilosomes (B9) showing scattered perfect spherical particles with no aggregations.

Figure 3 shows the morphological appearance of the selected RES-loaded bilosomes as examined using TEM, where the micrographs show scattered perfect spherical particles with no aggregations.

### 3.4. Behavioral Assessment

#### 3.4.1. Y-Maze Test

**Figure 4:**
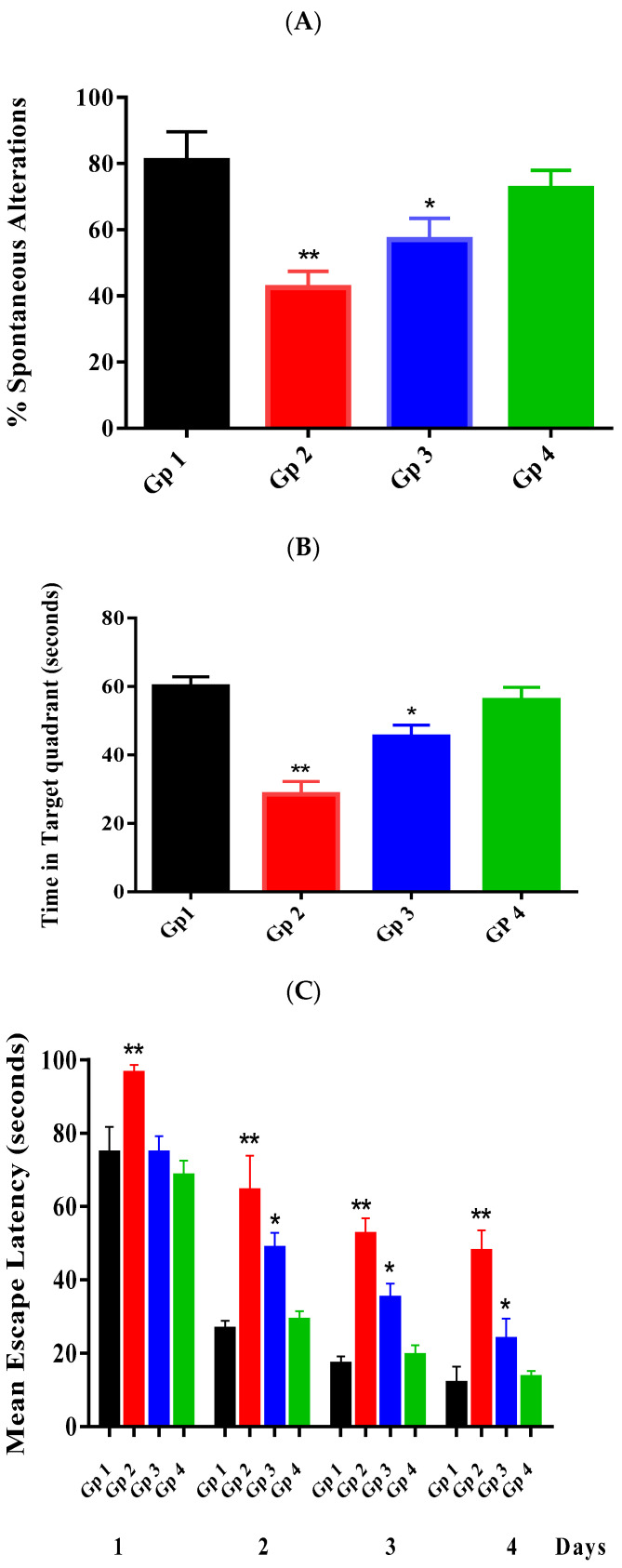
Effect of RES on the % spontaneous alterations of the Y-maze test (**A**), time spent in the target quadrant (**B**) in the MWM test, the escape latency (**C**) in the MWM test. Statistical analyses of the mean percentage of results were performed using one-way analysis of variance (ANOVA) (*p* < 0.05), followed by the Tukey–Kramer post hoc test, whereby each value was presented as mean ± standard deviation of the mean, where (*) and (**) indicate statistically different values from the control group at (*p* < 0.05, and *p* < 0.01), respectively.

Figure 4A shows the % spontaneous alterations recorded for different groups after performing the Y-maze test. The % spontaneous alterations recorded for different groups show statistically significant (*p* < 0.05) differences between groups except for Gp 1 and Gp 4.

As revealed from Gp 2, injection of STZ successfully impaired the learning and the memory abilities of the injected mice. This was manifested by a significant (*p* < 0.05) decrease in the % spontaneous alterations of Gp 2 compared to the normal group (Gp 1). This may be attributed to the toxic effect of STZ on the insulin-producing beta-cells in the CNS with subsequent impairment in the cholinergic system. This effect results in a remarkable decrease in choline acetyltransferase activity and a significant increase in acetylcholinesterase (AChE) activity. In addition to its effect on reducing hippocampal synaptic transmission, behavioral and pathological symptoms resemble AD [32,33]. Moreover, it has been reported that insulin is responsible for stimulating the extracellular Aβ secretion and inhibiting its degradation [34]. The obtained results are following previous studies that reported the effect of STZ on the behavior of the injected mice [24,35].

As revealed, both Gps 3 and 4 showed a significant (*p* < 0.05) increase in the % spontaneous alterations compared to Gp 2, indicating the ability of RES to improve the learning and the memory abilities of the injected mice. However, only RES-loaded bilosomes showed a non-significant (*p* ˃ 0.05) difference from the normal group compared to RES suspension that was unable to restore the values of the % spontaneous alterations to normal levels. The obtained results indicate the effectiveness of RES-loaded bilosomes in treating AD compared to RES suspension. RES-loaded bilosomes’ higher efficiency may be attributed to their enhanced absorption from the GIT, hence increasing RES bioavailability.

#### 3.4.2. Morris Water Maze Test

Figure 4B shows the time spent by each group to locate the place of the removed platform, where the mean time (56 s) spent in the target quadrant for mice treated with RES bilosomes (Gp 4) was doubled as compared to that recorded (28 s) for the negative control (Gp 2) with a non-significant difference from the normal group (60 s) (Gp 1). The group receiving RES suspension (Gp 3) also spent a significantly longer time (45 s) in the target quadrant than that measured for Gp 2, which was significantly shorter than that of the normal group.

Figure 4C shows the results of the MEL, which ensured the effect of injection of STZ (Gp 2) on the impairment of the learning and the memory abilities of the injected mice. As revealed, the values of the MEL were significantly (*p* < 0.05) different among the investigated groups. Gp 1, representing the normal mice, had a significant tendency to escape as presented by the low value of the latency time of 11.8 ± 4.12 s on a day in contrast to Gp 2 that shows a significant (*p* < 0.05) delay in the escaping tendency, with a value of 47.8 ± 5.11 s. This tendency may be attributed to the enhanced effect of SZT on the memorial ability.

As observed, Gps 3 and 4 treated with RES suspension and RES-loaded bilosomes, respectively, showed a significant (*p* < 0.05) decrease in the MEL time (23.8 ± 5.03 and 13.4 ± 1.62 respectively) in comparison to Gp 2. However, only Gp 4 showed a non-significant (*p* ˃ 0.05) difference as compared to Gp 1, indicating the ability of RES-loaded bilosomes to restore the MEL time to the normal level, which may be assigned to the enhanced improvement of RES release from bilosomes in comparison with drug suspension with subsequent increase in drug absorption.

The obtained results indicate the significant effect of RES on memory enhancement. Our results follow previous studies that reported the impact of RES on elevating the brain-derived neurotrophic factor and improving the damaged hippocampal circuits that restore cognition and memory loss [36,37]. Finally, results of the MWM test indicate the ability of RES to repair memory loss in STZ induced mice model [38,39].

### 3.5. Neurochemical Assay

As previously mentioned, AD being a neurodegenerative disease is accompanied by many elevated levels of oxidative stress and inflammatory biomarkers. It has been proposed that COX2 is involved in the cascade of events leading to AD; consequently, its expression is particularly elevated in AD brains and is closely related to dementia. In addition, it has been suggested that IL-1β is regulated by COX2 and, therefore, treatment involving COX2 inhibition is involved in AD [5]. Previous studies described the role of both COX2 and IL-6 in AD due to their involvement in the inflammatory response with subsequent increase in neurons damage [5,40,41].

Based on the above, the two biomarkers (COX2 and IL-6) were chosen to study the effect RES on the regulation of oxidative stress in AD brains. Table 2 shows the results of the neurochemical assay of the pro-inflammatory biomarkers (IL-6 and COX2) in the brain of the different groups. Gp 1 (normal mice) showed the lowest normal levels of the measured biomarkers, which are significantly (*p* < 0.05) lower than those obtained from other groups. Gp 2 showed about 3.5-fold and 3.9-fold increase in IL-6 and COX2, respectively, compared to Gp 1. Moreover, Gps 3 and 4 that received RES suspension and RES-loaded bilosomes, respectively, showed significantly (*p* < 0.05) lower levels than Gp 2, indicating their ability to reduce the inflammatory reaction.

It has been reported that RES can moderate the downregulation of several pro-inflammatory biomarkers such as COX2 and IL-6; hence, RES plays an important role in the process of neuroinflammation. In addition, RES is capable of decreasing oxidative stress either directly or indirectly. It acts directly by neutralizing and scavenging the ROS, decreasing the activity of enzymes elaborated in OS formation. Meanwhile, it acts indirectly by overexpressing genes responsible for antioxidant enzymes and suppressing genes encoding for OS proteins [8]. Regulation of inflammatory biomarkers is accompanied by improvement in memory and behavior.

Table 2 shows that the normal level of Aβ and Tau proteins in normal mice (Gp 1) are significantly (*p* < 0.05) different from other groups, where Gp 2 showed the highest levels among all groups. Gps 3 and 4 show significantly (*p* < 0.05) lower levels than Gp 2. It has been reported that there is a mutual effect between Aβ1-42 and IL-6, where IL-6 stimulates the production of the β-amyloid precursor protein (APP), and contrarily, IL-6 is upregulated upon stimulation with the carboxy-terminal 105 amino acids of APP, enhancing the neuronal damage induced by Aβ1-42 [40].

Moreover, the ubiquitin–proteasome system (UPS), the primary proteolytic mechanism responsible for degrading proteins including Aβ and p-Tau, is impaired in AD brains [42]. RES could maintain proteins control by regulating UPS function [43]. In addition, RES encourages Aβ clearance by inhibiting Aβ fibril formation, accumulation and deposition, and restoring the normal cellular autophagy, and reduces the Tau protein level by promoting its degradation and deacetylation.

All behavioral and biochemical results indicate the effectiveness of RES-loaded bilosomes. The obtained results correlate well with the in vitro release study that demonstrates the ability of bilosomes to completely release the drug with a subsequent increase in drug dissolution and bioavailability.

### 3.6. Histological Examination Results

**Figure 5:**
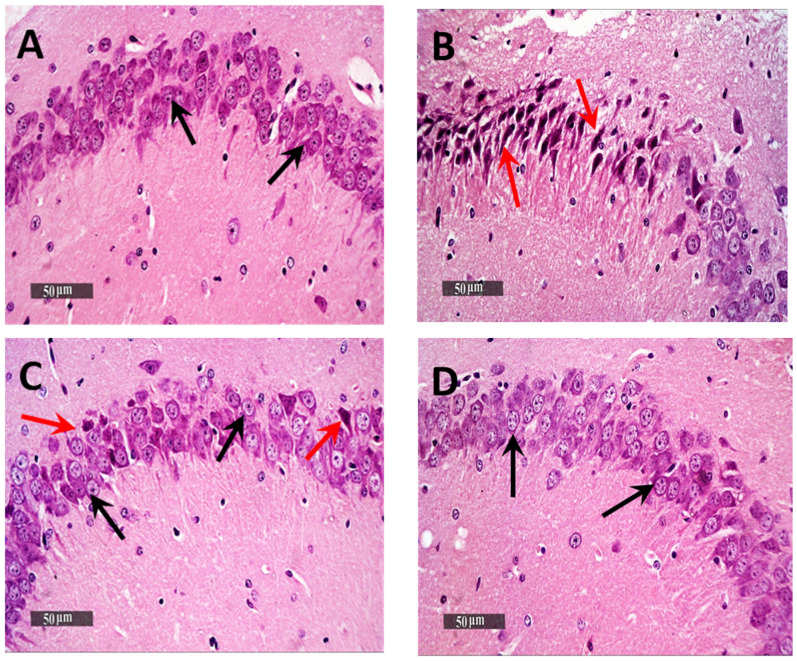
Neuroprotective histological effect of different treatments on CA3/hippocampal sub-regions of AD model. (**A**) normal group, (**B**) negative control group (received 0.9% saline), (**C**) positive control group (received resveratrol suspension) and (**D**) test group (received resveratrol bilosomes). H&E stain, 400×.

**Figure 6:**
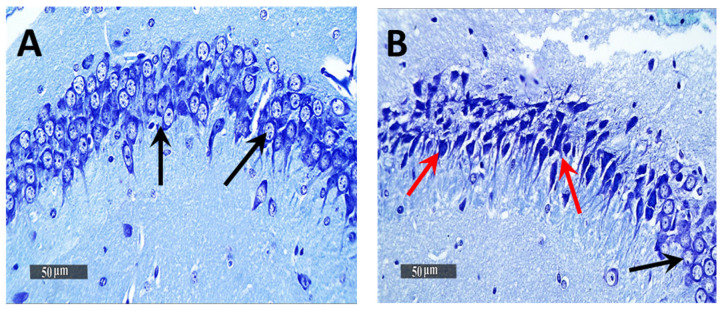

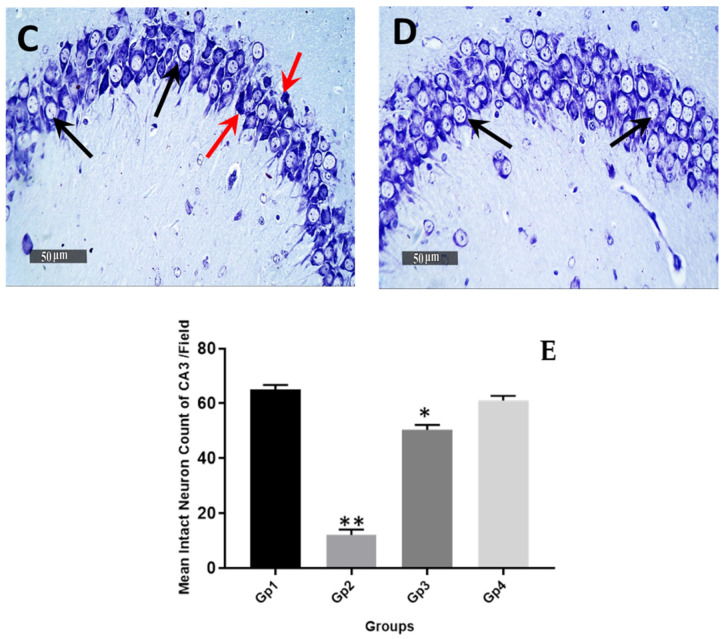
Light-microscopic examination of toluidine blue-stained pyramidal neurons in CA3/hippocampal sub-regions in different groups. (**A**) normal group, (**B**) negative control group (received 0.9% saline), (**C**) positive control group (received resveratrol suspension) and (**D**) test group (received resveratrol bilosomes). 400×. Black arrows = intact neurons, Red arrows = damaged neurons. The bar chart (**E**) shows the mean ± SD of the intact neuron count of CA3 in different groups where Gp 1 represents the normal group, Gp 2, Gp 3 and Gp 4 represent the negative control group (received 0.9% saline), positive control group (received resveratrol suspension) and test group (received resveratrol bilosomes), respectively, where (*) and (**) indicate statistically significantly different values from the control group at (*p* < 0.05 and *p* < 0.01), respectively.

Light Microscopic examination of the hippocampal subregion in normal control samples demonstrated normal morphological features of hippocampal CA3 and dentate gyrus regions. The CA3 subregion showed well organized cellular elements, including 5–6 cells thick pyramidal neurons middle layer with apparent intact subcellular details (Figure 5A—black arrows) (mean intact pyramidal neurons count in Toluidine blue-stained sections = 65 cells/field) (Figure 6A). Intact neuropil with normally distributed glial cells and normal vasculatures were observed. However, the AD-induced model samples CA3 subregion showed severe neuronal damage with abundant records of degenerated, hypereosinophilic and shrunken neurons losing their subcellular details (Figure 5B—Red arrows) accompanied with moderate perineuronal oedema and higher records of astrogliosis as well as microglial cells infiltrates (Figure 5B—arrowhead). The mean intact neurons count was 12 cells/field in Toluidine blue-stained tissue sections (Figure 6B). Gp 3 samples demonstrated significant neuroprotective efficacy with many apparent intact neurons (Figure 5C—black arrows) and few sporadic neuronal degenerative changes records (Figure 5C—Red arrows) with mean intact neurons count = 50 cells/field in Toluidine blue-stained tissue sections (Figure 6C). Mild persistence records of astrogliosis or microglial cell infiltrate were shown. Moreover, Gp 4 samples showed well organized morphological features of CA3 regions with almost apparent intact neurons (Figure 5D—black arrows) and minimal records of abnormal glial cells infiltrates or oedema, with mean intact cells count = 61 cells/field respectively in Toluidine blue-stained tissue sections (Figure 6D).

**Figure 7:**
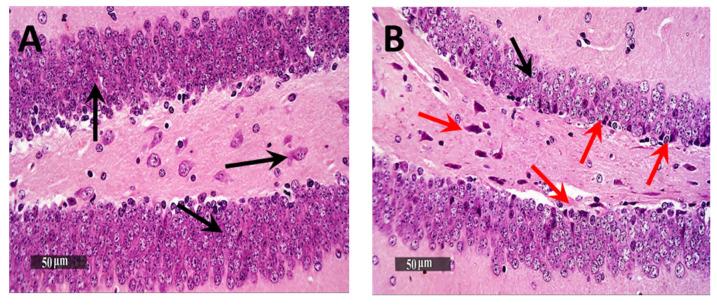

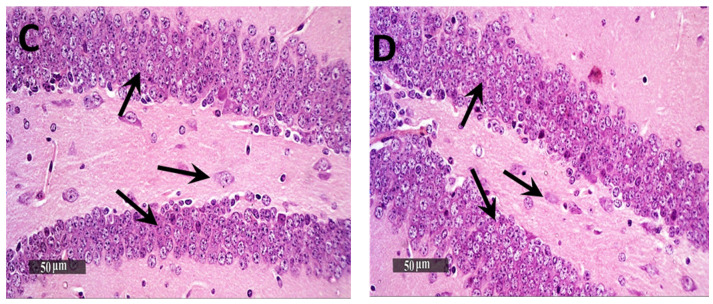
Neuroprotective histological effect of different treatments on Dentate Gyrus/hippocampal sub-regions of AD model. (**A**) Normal group, (**B**) Negative control group, (**C**) Positive control group and (**D**) Test group. H&E stain, 400×.

**Figure 8:**
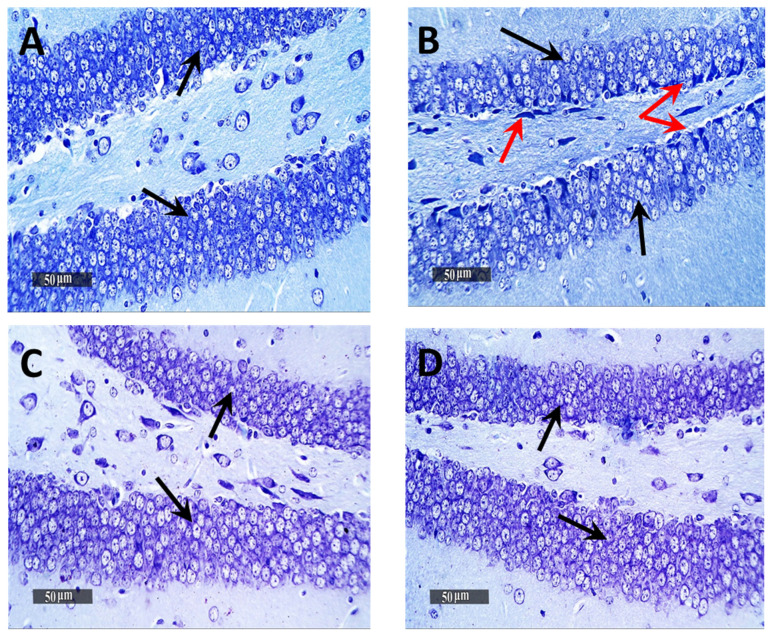

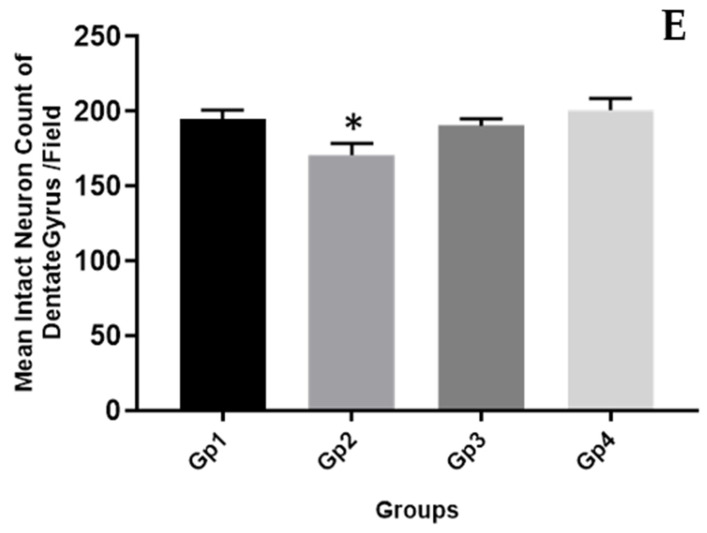
Light-microscopic examination of toluidine blue-stained pyramidal neurons in Dentate Gyrus/hippocampal subregions in different groups. (**A**) Normal group, (**B**) Negative control group, (**C**) Positive control group and (**D**) Test group 400×, Black arrows = intact neurons, Red arrows = damaged neurons. The bar chart (**E**) shows the mean ± SD of the intact neuron count of Dentate Gyrus, where Gp 1 represents the normal group, Gp 2, Gp 3 and Gp 4 represent the negative control group (reveived 0.9% saline), positive control group (received resvertrol suspension) and test group (received resveratrol bilosomes), respectively. * indicates statistically signifcantly different values from the control group (Gp 1) at *p* < 0.05.

Microscopic examination of Dentate Gyrus sub-regions of normal control samples demonstrated many apparent intact well-organized granule cells neurons all over Dentate Gyrus blades (Figure 7A—black arrows) with mean intact granule cells count = 195 cells/field in Toluidine blue-stained tissue sections (Figure 8A). Moreover, almost intact hilar cells in the hilus region were observed. On the other hand, the Dentate Gyrus region of model samples showed moderate records of nuclear pyknosis and degenerative neuronal changes of inner small granule cells layer as well as hilar cells damage and loss (Figure 7B), with mean intact granule cells count = 170 cells/field (Figure 8B). Obvious neuroprotective efficacy was shown in Gps 3 and 4 subregions in different samples (Figure 7C,D, respectively), with almost the same records of mean intact granule cells counts observed resembling normal control samples, as well as almost intact hilar cells (Figure 8C,D). In addition, minimal sporadic records of degenerative changes in granule cells were observed.

### 3.7. Immuno-Histochemical Analysis

**Figure 9:**
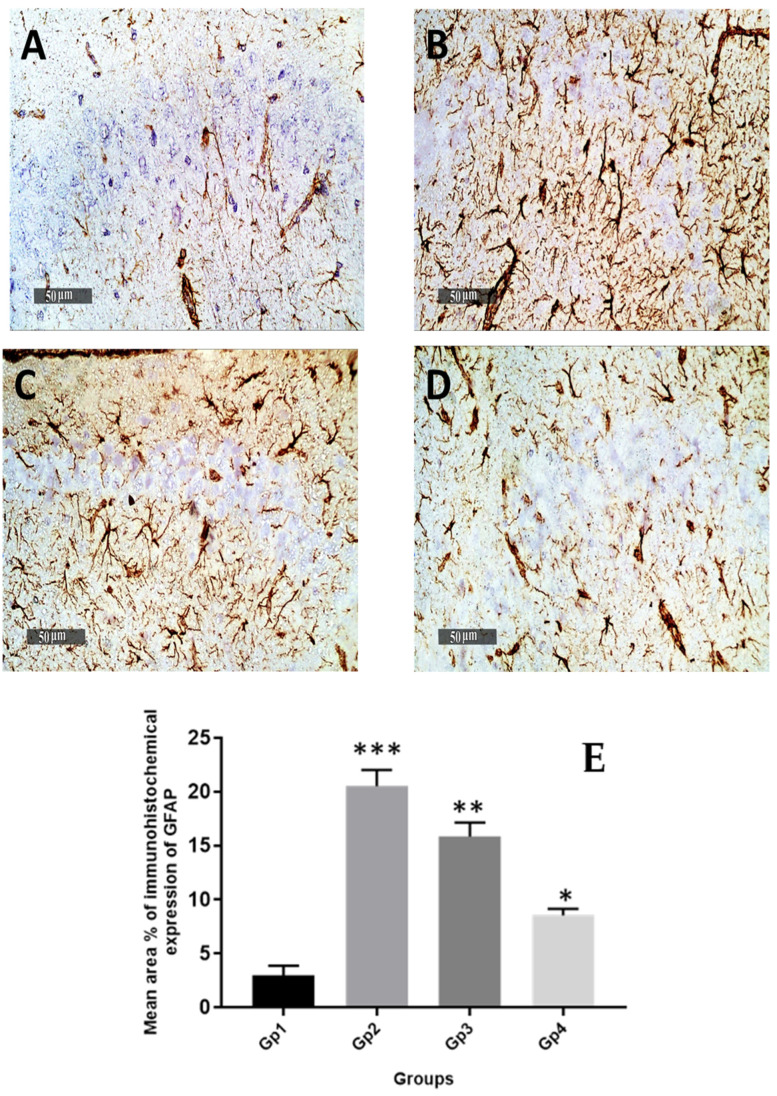
Effect of different treatments on hippocampal GFAP immuno-histochemical levels in reactive astrocytes expressed as the mean area percentage of expression. (**A**) Normal group, (**B**) Negative control group, (**C**) Positive control group and (**D**) Test group. 400×. The bar chart (**E**) shows the mean ± SD of the % area of immuno-histochemical levels expression of GFAP, where Gp 1 represents the normal group, Gp 2, Gp 3 and Gp 4 represent the negative control group (received 0.9% saline), positive control group (received resveratrol suspension) and test group (received resveratrol bilosomes), respectively, where (*), (**) and (***) indicate statistically significantly different values from the control group at (*p* < 0.05, *p* < 0.01 and *p* < 0.001), respectively.

**Figure 10:**
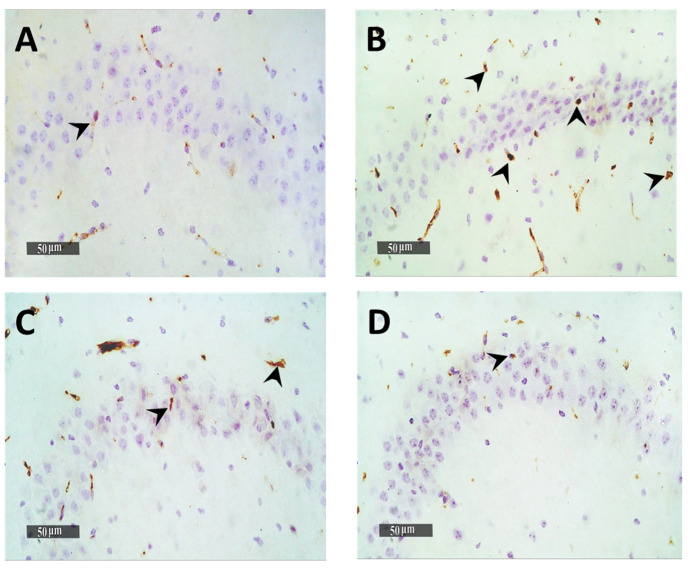

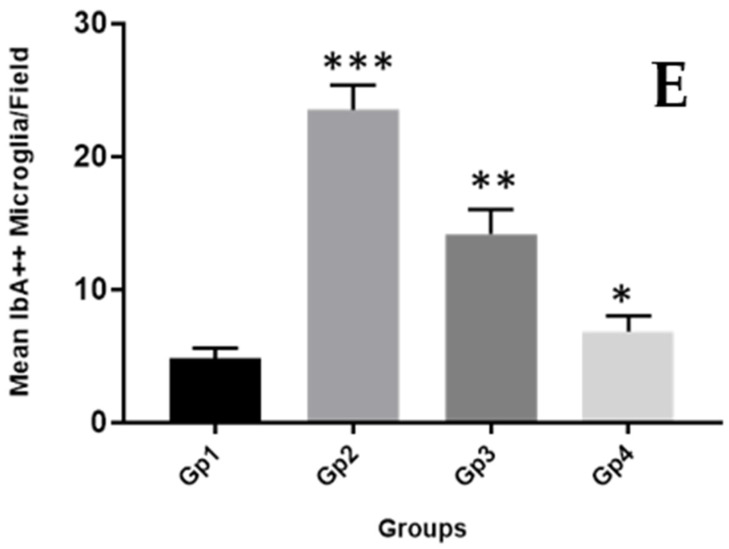
Effect of different treatments on hippocampal IbA/++ activated microglial cells count expressed as the mean number of IbA/++ cells per microscopic field. (**A**) Normal group, (**B**) Negative control group, (**C**) Positive control group and (**D**) Test group. 400×. The bar chart (**E**) shows the mean ± SD of the % area of immuno-histochemical levels expression of GFAP, where Gp 1 represents the normal group, Gp 2, Gp 3 and Gp 4 represent the negative control group (received 0.9% saline), positive control group (received resveratrol suspension) and test group (received resveratrol bilosomes), respectively, where (*), (**) and (***) indicate statistically significantly different values from the control group at (*p* < 0.05, *p* < 0.01 and *p* < 0.001), respectively.

**Figure 11:**
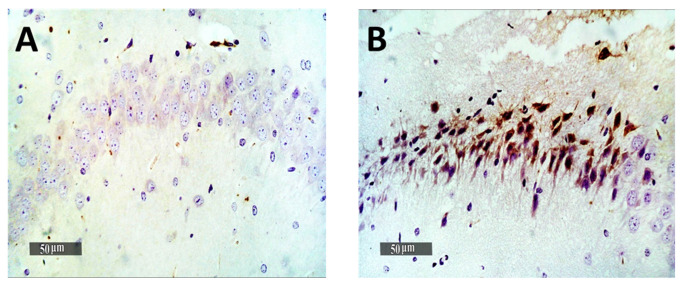

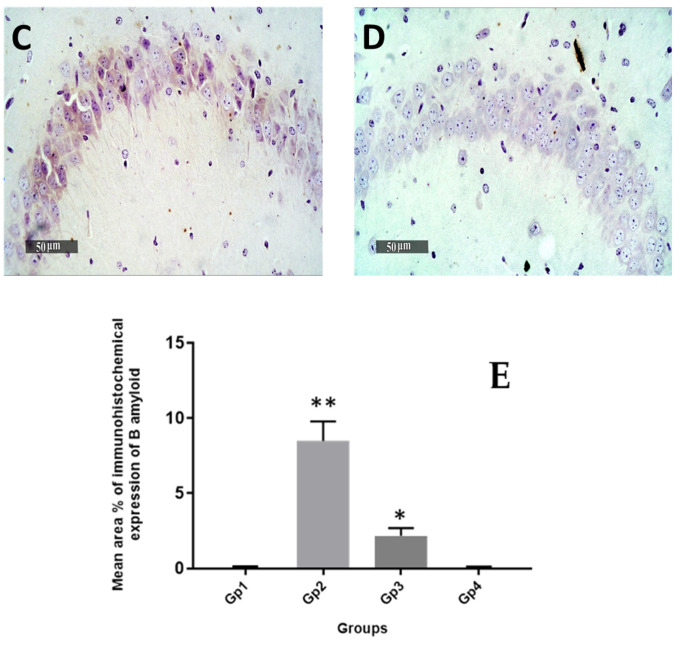
Effect of different treatments on hippocampal intraneuronal Amyloid B immunohistochemical levels in pyramidal neurons expressed as the mean area percentage of expression. (**A**) Normal group, (**B**) Negative control group, (**C**) Positive control group and (**D**) Test group. 400×. The bar chart (**E**) shows the mean ± SD of the % area of immuno-histochemical levels expression of ß-Amyloid, where Gp 1 represents the normal group, Gp 2, Gp 3 and Gp 4 represent the negative control group (received 0.9% saline), positive control group (received resveratrol suspension) and test group (received resveratrol bilosomes), respectively, where (*) and (**) indicate statistically significantly different values from the control group at (*p* < 0.05 and *p* < 0.01), respectively.

Quantitative analysis of GFAP immuno-histochemical expression in different groups showed that the mean expression levels in normal control samples were nearly 2.9%. However, it was observed a 7-fold increase of mean GFAP expression levels in model induced samples. The results revealed a significant (*p* < 0.05) decrease of mean expression values up to 23% and 58.6% in Gps 3 and 4 treated samples, respectively, compared with mean model induced group samples (Figure 9). Obvious higher records of activated IbA1/++ microglial cells count up to 4.8% folds increase in model induced samples compared with normal control samples records were obtained. Moreover, significant lower signaling of activated IbA1/++ microglial cells count was recorded in Gp 3 treated samples compared with model samples (up to 40% compared with model samples) (Figure 10). Among all treatment groups, the minimal observed mean quantitative records of activated IbA1/++ microglial cells count was recorded in Gp 4 treatment samples without significant difference compared with normal control samples. Intraneuronal/++ β-amyloid mean records showed up to 8.4% of AD induced model samples hippocampal neurons with positive immunoexpressions. However, a significant reduction up to 74.2% was observed in Gp 3 samples. Moreover, the Gp 4 samples showed nearly the same scanty records as the normal control samples (Figure 11).

## 4. Conclusions

Bilosomes were successfully prepared using the thin-film hydration method and optimized by varying different factors such as the number of extrusion cycles, drug concentration, pH of the medium and the effect of cholesterol addition. The studied factors had a significant effect on the physicochemical properties of RES-loaded bilosomes. B9 showed the highest %EE of the drug, and a favorable size and fastest drug release were selected for further study. TEM micrographs showed scattered perfect spherical particles with no aggregations. All behavioral results of Morris water and Y-maze tests indicate the effectiveness of RES-loaded bilosomes as a memory enhancer. RES-loaded bilosomes were able to decrease the oxidative stress biomarkers (IL-6 and COX2) and proteins (Tau and ß-amyloid levels that play an important role in Alzheimer disease. The obtained results correlate well with the in vitro release study that demonstrates the ability of bilosomes to completely release the drug with a subsequent increase in drug dissolution and bioavailability. Finally, RES-loaded bilosomes showed superiority over RES suspension as demonstrated by the histological examination of mice brains and the immuno-histochemical analysis. Therefore, RES-loaded bilosomes could be considered as an efficient platform for the treatment of Alzheimer disease.

## Figures and Tables

**Table 1:** The composition and physicochemical characterization of different RES-loaded bilosomes.

Formula	Molar Ratio of SPC/SDC/CH	Number of Extrusion Cycles	Drug Conc. (mg/mL)	pH	%EE	Particle Size (nm)	PDI	Zeta Potential (mV)
B1	4:1:0	1	5	7.4	16.3 ± 1.56	254 ± 1.47	0.621 ± 0.011	−31.0 ± 1.65
B2	4:1:0	2	5	7.4	19.3 ± 1.20	218 ± 1.95	0.745 ± 0.012	−29.0 ± 2.06
B3	4:1:0	3	5	7.4	29.3 ± 2.05	198 ± 1.45	0.512 ± 0.016	−26.6 ± 1.08
B4	4:1:0	4	5	7.4	21.2 ± 0.98	210 ± 2.05	0.806 ± 0024	−27.2 ± 1.45
B5	4:1:0	3	10	7.4	67.0 ± 1.56	316 ± 1.87	0.330 ± 0.022	−28.0 ± 1.52
B6	4:1:0	3	20	7.4	54.0 ± 2.52	375 ± 2.08	0.723 ± 0.031	−27.0 ± 2.23
B7	4:1:0	3	30	7.4	42.0 ± 1.96	381 ± 2.60	0.780 ± 0.018	−26.4 ± 1.78
B8	4:1:0	3	10	3	72.3 ± 1.87	265 ± 1.58	0.18 ± 0.013	−35.5 ± 2.80
B9	4:1:1	3	10	3	76.2 ± 1.36	189 ± 2.14	0.116 ± 0.015	−31.2 ± 2.2

SPC: LIPOID S100 (soybean phosphatidylcholine), SDC: sodium deoxycholate and CH: Cholesterol, %EE: entrapment efficiency, PDI: polydispersity index. Each result is the mean of three determinations ± SD.

**Table 2:** Results of neurochemical assay of different biomarkers measured after homogenization of mice brain obtained from different groups.

Groups	IL-6 Pg/g Tissue	COX2 Pg/g Tissue	Amyloid Beta pg/g Tissue	Tau ng/g Tissue
Group 1 (normal mice)	9.8 ± 0.85	6.8 ± 1.14	8.2 ± 0.76	7.4 ± 1.6
Group 2 (negative control)	34.2 ± 1.16	26.5 ± 1.05	28.7 ± 1.69	30.1 ± 1.08
Group 3 (positive control)	28.9 ± 1.3	20.4 ± 1.6	24.6 ± 1.2	25.1 ± 0.87
Group 4 (test group)	16.5 ± 1.75	12.6 ± 0.65	14.6 ± 1.25	13.9 ± 1.05

Animals (*n* = 8) in each group were subjected to the neurobehavioral tests. Statistical analyses of mean concentrations were performed using one-way analysis of variance (ANOVA) followed by the Tukey–Kramer post hoc test, whereby each value was presented as mean ± standard deviation of the mean (*p* < 0.05). All groups were statistically significantly different from the normal control group.

## Data Availability

All data are reported in the manuscript.

## References

[1] Imbimbo B.P., Lombard J., Pomara N. (2005). Pathophysiology of Alzheimer’s Disease. Neuroimaging Clin. N. Am..

[2] Wen M.M., El-Salamouni N.S., El-Refaie W.M., Hazzah H.A., Ali M.M., Tosi G., Farid R.M., Blanco-Prieto M.J., Billa N., Hanafy A.S. (2017). Nanotechnology-based drug delivery systems for Alzheimer’s disease management: Technical, industrial, and clinical challenges. J. Control. Release.

[3] Petersen S.K., Smith C. (2016). Ageing-Associated Oxidative Stress and Inflammation Are Alleviated by Products from Grapes. Oxid. Med. Cell. Longev..

[4] Danta C.C., Piplani P. (2014). The discovery and development of new potential antioxidant agents for the treatment of neurodegenerative diseases. Exp. Opin. Drug Discov..

[5] Wang P., Guan P.-P., Wang T., Yu X., Guo J.-J., Wang Z.-Y. (2014). Aggravation of Alzheimer’s disease due to the COX-2-mediated reciprocal regulation of IL-1β and Aβ between glial and neuron cells. Aging Cell.

[6] Sahni J.K., Doggui S., Ali J., Baboota S., Dao L., Ramassamy C. (2011). Neurotherapeutic applications of nanoparticles in Alzheimer’s disease. J. Control. Release.

[7] Shamarekh K.S., Gad H.A., Soliman M.E., Sammour O.A. (2020). Development and evaluation of protamine-coated PLGA nanoparticles for nose-to-brain delivery of tacrine: In-vitro and in-vivo assessment. J. Drug Deliv. Sci. Technol..

[8] Griñán-Ferré C., Bellver-Sanchis A., Izquierdo V., Corpas R., Roig-Soriano J., Chillón M., Andres-Lacueva C., Somogyvári M., Sőti C., Sanfeliu C. (2021). The pleiotropic neuroprotective effects of resveratrol in cognitive decline and Alzheimer’s disease pathology: From antioxidant to epigenetic therapy. Ageing Res. Rev..

[9] Labban S., Alghamdi B.S., Alshehri F.S., Kurdi M. (2021). Effects of melatonin and resveratrol on recognition memory and passive avoidance perfor-mance in a mouse model of Alzheimer’s disease. Behav. Brain Res..

[10] Guan P., Lu Y., Qi J., Wu W. (2016). Readily restoring freeze-dried probilosomes as potential nanocarriers for enhancing oral delivery of cyclosporine A. Colloids Surf. B Biointerfaces.

[11] Al-Edresi S., Alsalahat I., Freeman S., Aojula H., Penny J. (2020). Resveratrol-mediated cleavage of amyloid β1–42 peptide: Potential relevance to Alzheimer’s disease. Neurobiol. Aging.

[12] Komorowska J., Wątroba M., Szukiewicz D. (2020). Review of beneficial effects of resveratrol in neurodegenerative diseases such as Alzheimer’s disease. Adv. Med. Sci..

[13] Loureiro J.A., Andrade S., Duarte A., Neves A.R., Queiroz J.F., Nunes C., Sevin E., Fenart L., Gosselet F., Coelho M.A. (2017). Resveratrol and Grape Extract-loaded Solid Lipid Nanoparticles for the Treatment of Alz-heimer’s Disease. Molecules.

[14] Yang L., Wang W., Chen J., Wang N., Zheng G. (2018). A comparative study of resveratrol and resveratrol-functional selenium nanoparticles: Inhibiting amyloid β aggregation and reactive oxygen species formation properties. J. Biomed. Mater. Res. Part A.

[15] Frozza R.L., Bernardi A., Hoppe J.B., Meneghetti A.B., Matté A., Battastini A.M., Pohlmann A.R., Guterres S.S., Salbego C. (2013). Neuroprotective effects of resveratrol against Aβ administration in rats are improved by lipid-core nanocapsules. Mol. Neurobiol..

[16] Salem H.F., Kharshoum R.M., Abou-Taleb H.A., Naguib D.M. (2019). Brain targeting of resveratrol through intranasal lipid vesicles labelled with gold nanoparticles: In vivo evaluation and bioaccumulation investigation using computed tomography and histopathological examination. J. Drug Target..

[17] da Rocha Lindner G., Bonfanti Santos D., Colle D., Gasnhar Moreira E.L., Daniel Prediger R., Farina M., Khalil N.M., Mara Mainardes R. (2015). Improved neuroprotective effects of resveratrol–loaded polysorbate 80-coated poly(lactide) nanoparticles in MPTP-induced Parkinsonism. Nanomedicine (Lond).

[18] Lu X., Ji C., Xu H., Li X., Ding H., Ye M., Zhu Z., Ding D., Jiang X., Ding X. (2009). Resveratrol-loaded polymeric micelles protect cells from Abeta-induced oxidative stress. Int. J. Pharm..

[19] Shukla A., Mishra V., Kesharwani P. (2016). Bilosomes in the context of oral immunization: Development, chal-lenges and opportunities. Drug Discov. Today.

[20] Elnaggar Y.S.R., Omran S., Hazzah H.A., Abdallah O.Y. (2019). Anionic versus cationic bilosomes as oral nanocarriers for enhanced delivery of the hydrophilic drug risedronate. Int. J. Pharm..

[21] Chen Y., Lu Y., Chen J., Lai J., Sun J., Hu F., Wu W. (2009). Enhanced bioavailability of the poorly water-soluble drug fenofibrate by using liposomes con-taining a bile salt. Int. J. Pharm..

[22] Mansour M., Abo El Ezz T.A., Fattoh F.N., AbouelFadl D.M., Gad H.A. (2021). Delineating the usage of Dexamethasone-loaded cubosomes as a therapeutic armamentarium for hearing loss versus its protective effect: In vitro and in vivo animal study. J. Drug Deliv. Sci. Technol..

[23] Hathout R.M., Gad H.A., Metwally A.A. (2017). Gelatinized-core liposomes: Toward a more robust carrier for hydrophilic molecules. J. Biomed. Mater. Res. Part A.

[24] Sorial M.E., el Sayed N. (2017). Protective effect of valproic acid in streptozotocin-induced sporadic Alzheimer’s disease mouse model: Possible involvement of the cholinergic system. Naunyn Schmiedebergs Arch. Pharmacol..

[25] Yamada K., Tanaka T., Mamiya T., Shiotani T., Kameyama T., Nabeshima T. (1999). Improvement by nefiracetam of beta-amyloid-(1-42)-induced learning and memory impairments in rats. Br. J. Pharmacol..

[26] Culling C.F.A. (2013). Handbook of Histopathological and Histochemical Techniques.

[27] Hu S., Niu M., Hu F., Lu Y., Qi J., Yin Z., Wu W. (2013). Integrity and stability of oral liposomes containing bile salts studied in simulated and ex vivo gastrointestinal media. Int. J. Pharm..

[28] Sun J., Deng Y., Wang S., Cao J., Gao X., Dong X. (2010). Liposomes incorporating sodium deoxycholate for hexamethylmelamine (HMM) oral delivery: Development, characterization, and in vivo evaluation. Drug Deliv..

[29] Gad H.A., Kamel A.O., Sammour O.A., El Dessouky H.F. (2013). Vesicular powder as carrier for doxycycline hydrochloride and metronidazole combination therapy. Pharm. Dev. Technol..

[30] Robinson K., Mock C., Liang D. (2015). Pre–formulation studies of resveratrol. Drug Dev. Ind. Pharm..

[31] Hathout R.M., Gad H.A., Abdel-Hafez S.M., Nasser N., Khalil N., Ateyya T., Amr A., Yasser N., Nasr S., Metwally A.A. (2019). Gelatinized core liposomes: A new Trojan horse for the development of a novel timolol maleate glaucoma medication. Int. J. Pharm..

[32] Agrawal R., Tyagi E., Shukla R., Nath C. (2009). A study of brain insulin receptors, AChE activity and oxidative stress in rat model of ICV STZ induced dementia. Neuropharmacology.

[33] Agrawal R., Tyagi E., Shukla R., Nath C. (2011). Insulin receptor signaling in rat hippocampus: A study in STZ (ICV) induced memory deficit model. Eur. Neuropsychopharmacol..

[34] Chesneau V., Vekrellis K., Rosner M.R., Selkoe D.J. (2000). Purified recombinant insulin-degrading enzyme degrades amyloid beta-protein but does not promote its oligomerization. Biochem. J..

[35] Liu P., Zou L.-B., Wang L.-H., Jiao Q., Chi T.-Y., Ji X.-F., Jin G. (2014). Xanthoceraside attenuates tau hyperphosphorylation and cognitive deficits in intracerebroventricular-streptozotocin injected rats. Psychopharmacology.

[36] Zhao Y.N., Li W.F., Li F., Zhang Z., Dai Y.D., Xu A.L., Qi C., Gao J.M., Gao J. (2013). Resveratrol improves learning and memory in normally aged mice through microRNA-CREB pathway. Biochem. Biophys. Res. Commun..

[37] Moorthi P., Premkumar P., Priyanka R., Jayachandran K.S., Anusuyadevi M. (2015). Pathological changes in hippocampal neuronal circuits underlie age-associated neurodegeneration and memory loss: Positive clue toward SAD. Neuroscience.

[38] Sharma M., Briyal S., Gupta Y.K. (2005). Effect of alpha lipoic acid, melatonin and trans resveratrol on intracerebroventricular streptozotocin induced spatial memory deficit in rats. Indian J. Physiol. Pharmacol..

[39] Sharma M., Gupta Y.K. (2002). Chronic treatment with trans resveratrol prevents intracerebroventricular streptozotocin induced cognitive impairment and oxidative stress in rats. Life Sci..

[40] Erta M., Quintana A., Hidalgo J. (2012). Interleukin-6, a Major Cytokine in the Central Nervous System. Int. J. Biol. Sci..

[41] Cojocaru I.M., Cojocaru M., Miu G., Sapira V. (2011). Study of interleukin-6 production in Alzheimer’s disease. Rom. J. Intern. Med..

[42] Xin S.-H., Tan L., Cao X., Yu J.-T., Tan L. (2018). Clearance of Amyloid Beta and Tau in Alzheimer’s Disease: From Mechanisms to Therapy. Neurotox. Res..

[43] Corpas R., Griñán-Ferré C., Rodríguez-Farré E., Pallàs M., Sanfeliu C. (2019). Resveratrol Induces Brain Resilience Against Alzheimer Neurodegeneration Through Proteo-stasis Enhancement. Mol. Neurobiol..

